# Effect of Alumina Nano-Particles on Physical and Mechanical Properties of Medium Density Fiberboard

**DOI:** 10.3390/ma13184207

**Published:** 2020-09-22

**Authors:** Hisham Alabduljabbar, Rayed Alyousef, Waheed Gul, Syed Riaz Akbar Shah, Afzal Khan, Razaullah Khan, Abdulaziz Alaskar

**Affiliations:** 1 Department of Civil Engineering, College of Engineering, Prince Sattam Bin Abdulaziz University, Alkharj 11942, Saudi Arabia; 2 Department of Mechanical Engineering, Institute of Space Technology, Islamabad 44000, Pakistan; 3 Department of Mechatronics Engineering, University of Engineering and Technology, Peshawar 25120, Pakistan; rasayed@uetpeshawar.edu.pk; 4 Department of Mechanical Engineering, University of Engineering and Technology, Peshawar 25120, Pakistan; afzalkhan@uetpeshawar.edu.pk; 5 Department of Mechanical Engineering Technology, University of Technology, Nowshera 24100, Pakistan; razaullah@uotnowshera.edu.pk; 6 Department of Civil Engineering, College of Engineering, King Saud University, Riyadh 12372, Saudi Arabia; Abalaskar@ksu.edu.sa

**Keywords:** composites, nano-composites, mechanical properties, tensile and compressive loads, process optimization

## Abstract

This research aims to explore the effects of nanoparticles such as alumina (Al_2_O_3_) on the physical and mechanical properties of medium density fiberboards (MDF). The nanoparticles are added in urea-formaldehyde (UF) resin with different concentration levels e.g., 1.5%, 3%, and 4.5% by weight. A combination of forest fibers such as *Populus Deltuidess* (Poplar) and *Euamericana* (Ghaz) are used as a composite reinforcement due to their exceptional abrasion confrontation as well as their affordability and economic value with Al_2_O_3_-UF as a matrix or nanofillers for making the desired nanocomposite specimens. Thermo-gravimetric analysis (TGA) and thermal analytical analysis (TAA) in the form of differential scanning calorimetry (DSC) are carried out and it has been found that increasing the percentage of alumina nanoparticles leads to an increase in the total heat content. The mechanical properties such as internal bonding (IB), modulus of elasticity (MOE) and modulus of rupture (MOR), and physical properties such as density, water absorption (WA), and thickness swelling (TS) of the specimens have been investigated. The experimental results showed that properties of the new Nano-MDF are higher when compared to the normal samples. The results also showed that increasing the concentration of alumina nanoparticles in the urea-formaldehyde resin effects the mechanical properties of panels considerably.

## 1. Introduction

Wood-based panels are manufactured with the help of heat-curing adhesive such as thermosetting resin which holds the wood fibers together. It has a potential for versatile designs and affordability. Also, the panels have long service life and better mechanical properties [1]. But the carcinogenic emission from the panel of formaldehyde is one of the disadvantages with the use of urea-formaldehyde resin [2]. To reduce formaldehyde emission, certain additives including formaldehyde catchers and melamine are used [3]. For the reduction of formaldehyde emission, Dudkin et al. [4], undertook the addition of aluminum oxide nanoparticles in urea-formaldehyde resin. The use of fibers in composites have many advantages including low cost, high quality, better mechanical properties, environmental friendliness, and reduced energy consumption [5]. Due to improved thermal, mechanical, and flammability properties, polymer-clay nano-composites have attracted attention. Recent laboratory experiments have shown that polymer-clay nano-composites provided improved mechanical properties and decreased flammability at low cost [6]. The properties of the polymeric composites are improved by nano-clays due to their special dimensions and high bonding capability with other fillers [7]. An increase in the demand of medium density fiberboard composite results in rapid economic growth of a country. Medium density fiberboard is a composite sheet formed under optimum pressure and temperature by the combination of wood or other plant fiber and urea-formaldehyde resin [8]. Composite material is mostly used in aerospace and automobile industry because of its light weight and high strength. However, the conventional production of composite materials make the use of ordinary structure which leads to poor physical and mechanical properties. Moreover, it emits formaldehyde which is harmful to health. Previous researches have been done to improve its strength using different concentration of nanoparticles in the urea-formaldehyde resin.

Gholamiyan et al. [9], studied the impact of polyester, nano-zycosil, nitrocellulose lacquer and acid catalyzed lacquer, nano-zycofil on contact angle and water absorption improvement. Based on T6E3 schedule, the poplar sapwood boards were dried. The boards were cut according to EN 927-5 standard and then immersed and coated with clear paints and nano particles. The samples of were then dried and the water absorption was measured for different immersion times. The results showed different water absorption pattern for nanoparticles and clear paints. It was concluded that samples coated with nano-zycosil and those coated with combined nitrocellulose lacquers and acid catalyzed lacquers has high resistance to water absorption. Also, the results showed greatest contact angle for nano-zycosil coated samples. Saraeian et al. [10], conducted a study with the main aim to find a proper proportion of nanoclay particles to increase flame retardancy and tensile strength of polystyrene-nanoclay composite. Nanocomposite samples were prepared from polystyrene and by an injection machine, the nanocomposite grains were then injected into a mold. Different tests were performed and the results revealed that with up to 5% increase in nanoclay, the modulus of elasticity and tensile strength increases while the strength decreases with 6% nanoclay. Also, the flame retardancy test results showed a decrease in heat release. Nemati et al. [11] studied nanoclay, recycled polystyrene and wood flour based mechanical properties of nanocomposites. For experimental purpose, the wood flour and recycled polystyrene were mixed at difference ratios. By the use of injection molding, wood plastic samples were prepared and bending and tensile tests were performed to evaluate mechanical properties. The results revealed that with the increase of nanoclay content in the composites, the flexural and tensile strengths increase. Taghiyari et al. [12], studied the effect of non-silver on the heat transfer rate to the central part of the medium density fiberboard. The experimental results showed that the uniform dispersion of nanoparticles all over the medium density fiberboard matrix considerably contributes to faster heat transfer to the central part. It was concluded that with the addition of metal nanoparticles for the purpose of increasing heat transfer may not necessarily improve all mechanical and physical properties. It was also concluded that metal particles’ optimum consumption level is dependent on several factors including coefficient of thermal conductivity of metal particles, hot press duration and hot press temperature. Taghiyari et al. [13], investigated the effects of wollastonite fibers on mechanical and physical properties of medium density fiberboards. The fiberboards selected for the study were comprises of wood fibers containing camel-thorn chips up to 10%. Results showed that most of the mechanical and physical properties were improved by wollastonite fibers. It was found that the addition of camel thorn chips affects the panel properties and 5% wollastonite fibers when combined with 10% camel-thorn produces panels with better properties. Ismaita et al. [14], inspected the effects of the addition of a filler of Cloisite Na^+^ to urea-formaldehyde resin on the mechanical and physical properties of particle boards. To evaluate the performance of the boards, thickness swelling, water absorption, density, internal bond strength, modulus of elasticity, and modulus of rupture were measured. Considerable improvements were noted in MOE, MOR and TS with the addition of Cloisite Na^+^ to the resin. Iždinský et al. [15], studied the effect of zinc-oxide and silver nanoparticles in acrylic coatings applied to timber composites, particleboard and medium density fiberboard. It was concluded from the study that antibacterial resistance of raw commercial wooden composites is higher if these composites contain a higher amount of formaldehyde. It was also concluded that antibacterial resistance of acryl-coated commercial wooden composites can be increased with a higher amount of zinc-oxide and silver nanoparticles. Due to contact with water, wood-based panels used in humid environments result in low durability. For increase in durability and fungi attack reduction, many studies developed resin with zinc oxide nanoparticles. To evaluate the physical properties, Silva et al. [16] aimed at the production of medium density fiberboard urea and melamine formaldehyde resins with the addition of 1.0% and 0.5% zinc oxide nanoparticles. It was found that with the addition of 1.0% of zinc nanoparticles resulted in higher moisture content and lower density panels. And the combination of 0.5% of zinc oxide nanoparticles with melamine-formaldehyde resulted in the best treatment of panels. Valle et al. [17], evaluated the impact of SiO_2_ nanoparticles on the mechanical and physical properties of wood particleboard. SiO_2_ nanoparticles were added to urea-formaldehyde to produce boards. When tested, results showed that the combination of SiO_2_ nanoparticles and adhesives of urea-formaldehyde increases the resistance to thickness swelling by 42% and hence improves the panel’s dimensional stability.

In many studies, various types of composites have been investigated. Barbosa et al. [18], and Kovarik et al. [19], studied particle reinforced composites while Jia et al. [20,21], worked on continuous fiber reinforced composites. Similarly, short fiber reinforced composites were studied by Yan et al. [22], and Dias et al. [23]. Different reinforcing fibers such as glass [24,25], carbon [20,21,25], and basalt [23,25,26], have usually been layered in 6 to 16 layers. Yan et al. [22], Kovarik et al. [19], and Bernal et al. [27], studied the mechanical properties of samples cured at high-temperature and laboratory temperature heat treatment. Some properties namely compressive strength [19,23,27], impact resistance [24,25], Young’s modulus [20,22,25], tensile properties [28,29], and flexural strength [19,20,21,22,25], were inspected on composites with alkali-activated aluminosilicates. It was found that the properties of composites with alkali-activated aluminosilicates depend on cure conditions of composites, type of fiber reinforcement, composition of alkali-activated aluminosilicates and number of layers of fiber reinforcement. Composites with carbon fabric and alkali-activated aluminosilicates prepared by Krystek et al. [29], were when tested, the tensile strength of the samples reached up to 265 MPa.

Though, the addition of nanoparticles has been analyzed in various contexts in literature; however, our study proposes specific nanoparticles called alumina (Al_2_O_3_) for the improvement of physical and mechanical properties of medium density fiberboard. Different concentrations of alumina nanoparticles were characterized for the optimization of the properties. When tested, considerable improvement in physical and mechanical properties was observed.

We have organized the reminder of this work as follows. The materials, methods and testing are explained in Section 2. In Section 3, the results are presented and discussed while Section 4 provides conclusion and future recommendations.

## 2. Materials and Methods

The materials and methods are illustrated in the subsections given below.

### 2.1. Materials

In this research work, the raw materials used are urea-formaldehyde resin, alumina nanoparticles, and natural fibers which are explained one by one in the subsections.

#### 2.1.1. Urea-Formaldehyde Resin

Urea-formaldehyde resin was provided by Wah Nobel Group of Companies, Taxila Pakistan. The specifications of the urea-formaldehyde resin can be seen in Table 1. The viscosity (cps), density (g/cc) and pH values were noted at a temperature of 25 °C, while the Gel time (seconds) was recorded at a temperature of 100 °C.

#### 2.1.2. Alumina Nanoparticles

Alumina nanoparticles were purchased from Guangzhou Hong Material Technology Company Limited, China. The reason of selection of alumina nanoparticles is due to its high heat transfer rate, hardness, insulation, and stability. The size of the particles ranges from 80 to 200 nanometers.

#### 2.1.3. Natural Fibers

The natural fibers *Populus Deltuidess* and *Euamericana* were provided by Ciel Woodworks Private Limited, Peshawar Pakistan. The average length of the natural fibers is 0.5 mm and it has 9% moisture content before its interaction with nanofillers.

### 2.2. Synthesis of Nanofluid

The Al_2_O_3_-UF nanofillers were prepared in the laboratory with the compositions given in Table 2 measured in grams.

**Figure 1:**
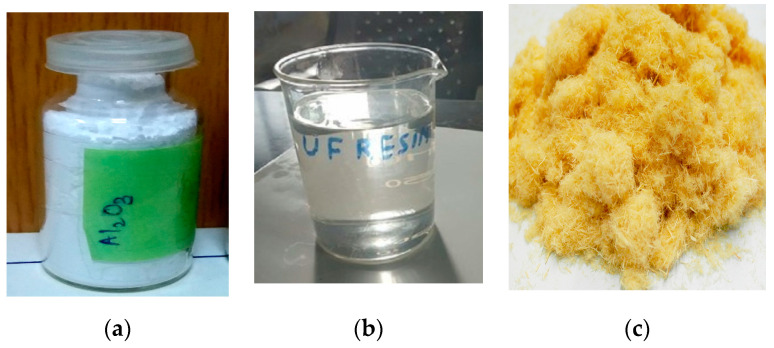
(**a**) Alumina nano-particles, (**b**) urea formaldehyde resin, and (**c**) natural fibers.

The nanofillers were synthesized by weighing 195 g of urea-formaldehyde resin and 3, 6, and 9 g of alumina nanoparticles of dry weight of fibers. Hence, in the four samples, the percentages Al_2_O_3_ in urea-formaldehyde resin are 0%, 1.54%, 3.08%, and 4.61%. The resin was then sonicated with Ultrasonic Processor UP 400S (Hielscher USA, Inc., Wanaque, NJ, USA) for 25 min. The samples were represented by Al_1_, Al_2_, Al_3_ and Al_4_ according to the concentration of alumina. The alumina nanoparticles, urea-formaldehyde resin, and natural fibers of *Populus Deltuidess* and *Euamericana* are depicted in Figure 1.

### 2.3. Composite Mix Design

The manufacturing process of medium density fiberboard starts with chipping of wood refined into fiber, mixed with glue and wax, and compressed under a high-temperature in a hot press. As a result, the glue heals, which is then bonded to the fiber to form medium density fiberboard.

The medium density fiberboard samples were prepared in panels with dimensions 450 into 450 into 16 mm by varying the density in the range 700–750 kg/m^3^. The Al_2_O_3_-UF nanofillers were sprayed on *Populus Deltuidess* and *Euamericana* fibers by means of spray gun. Hydraulic Hot Press (BURKLE, Bohemia, NY, USA) was used for hot pressing. The hot pressing process was carried out for all four samples at 185 °C temperature, 150 bar pressure of and for a duration of 4 min. The panels were allowed to cool horizontally in a cooling tower for 72 h after pressing.

### 2.4. Scanning Electron Microscopy of Alumina Nanoparticles

**Figure 2:**
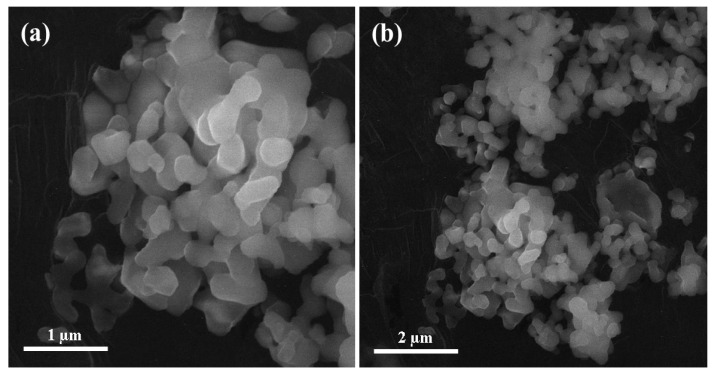
Scanning electron microscopy images of alumina nanoparticles at. (**a**) 50,000×, (**b**) 25,000×.

To perform scanning electron microscopy, a sample of alumina nanoparticles was prepared in the laboratory. The sample surface was coated with gold through Safematic CCU-010 Gold/Carbon Sputter (Labtech International Ltd., Heathfield, UK) before subjected to perform scanning electron microscopy. As shown in Figure 2, the scanning electron microscopy of alumina nanoparticles were examined through MIRA3 (TESCAN, Brno, Czech Republic) at magnifications of 50,000× and 25,000× with a maximum operating voltage of 10 kV. The selected alumina nanoparticles were almost spherical in shape and the size of the particles ranges from 80 to 200 nm.

### 2.5. Energy Dispersive X-Ray Spectroscopy (EDS) Analysis

Energy dispersive X-ray spectroscopy was performed through MIRA3 (TESCAN, Brno, Czech Republic) at magnifications of 50,000× and 25,000× with a maximum operating voltage of 10 kV on the area mapping of scanning electron microscopic images. This characterization was carried out to confirm the presence of alumina nanoparticles in the urea-formaldehyde resin.

### 2.6. Fourier Transform Infrared Spectroscopy (FTIR)

The Fourier Transform Infrared Spectroscopy of the urea-formaldehyde and Al_2_O_3_-UF resin was carried out through IR Prestige-21 Fourier Transform Infrared Spectroscopy (Shimadzu, Kyoto, Japan). The spectra were achieved in the spectral area 4000–500 cm^−1^ with a resolution of 2 cm^−1^ and 34 scans.

### 2.7. X-Ray Diffraction Analysis of Alumina Nanoparticles

**Figure 3:**
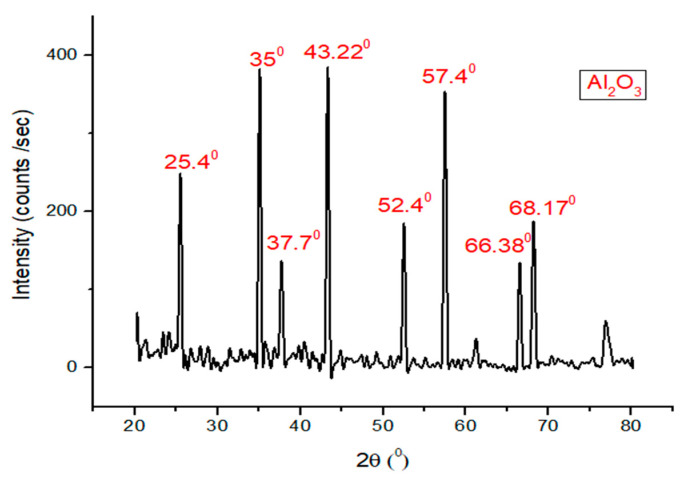
X-ray diffraction analysis of alumina nanoparticles.

X-ray diffraction analysis (Malvern Panalytical Ltd., Malvern, UK) of alumina nanoparticles was investigated. It was found that alumina shows peaks at 25.4°, 35°, 37.7°, 43.22°, 52.4°, 57.4°, 66.38°, and 68.170° as can be seen in Figure 3. The peak at 2θ equal to 35°, 43.22°, and 57.4° obtained for this sample are the strongest that can be compared with the peaks at 25.4°, 37.7°, 52.4°, 66.38°, and 68.17°.

### 2.8. Differential Scanning Calorimetry (DSC)

Differential Scanning Calorimetry was conducted using. The measurement was carried out between 20 °C and 180 °C with a heat rising rate of 10 °C/min in a Nitrogen flow of 10 mL/min.

### 2.9. Thermo-Gravimetric Analysis (*TGA*)

Thermo-gravimetric analysis was conducted using TGA/DSC-1-star system device (Mettler Toledo, Greifensee, Switzerland). The measurement was carried out between 25 °C and 800 °C with a heat rising rate of 10 °C/min in a Nitrogen flow of 10 mL/min.

### 2.10. Three-Point Bending Test

To evaluate the mechanical properties of the medium density fiberboard, Three-point Bending test was conducted. The samples size was 370 mm into 50 mm into 16mm according to EN-310 [30], with loading speed of 4 mm/min. The samples were tested using WDW-30 Electromechanical Universal Testing Machine of JINAN Precision Testing Equipment Company Limited, Jinan, China.

The mechanical properties were measured using Equations (1) and (2), respectively.

(1) $$\mathrm{Modulus}\;\mathrm{of}\;\mathrm{Elasticity}=\frac{PL^{3}}{4bd^{3}Y}$$

(2) $$\mathrm{Modulus}\;\mathrm{of}\;\mathrm{Rupture}=\frac{3PL}{2bd^{2}}$$

where, *P,* and *Y* are load and center deflection at proportional limit measured in N and mm, respectively. Also, *b*, *L* and *d* are width, length and depth of the samples measured in mm.

### 2.11. Tensile Strength (Internal Bonding) Test

Tensile strength test (Jinan Testing Equipment Corporation, Jinan, China) was performed perpendicular and parallel to the face of the specimen. A 50 mm into 50 mm specimen was glued with a bonding agent to steel or aluminum alloy of the similar sizes. Being a significant characteristic of composite board, internal bond strength was calculated using Equations (3) and (4).

(3) $$\mathrm{Internal}\;\mathrm{Bonding}=\frac{P}{bL}\;(\mathrm{Perpendicular}\;\mathrm{to}\;\mathrm{face})$$

(4) $$\mathrm{Internal}\;\mathrm{Bonding}=\frac{P}{bd}\;(\mathrm{Parallel}\;\mathrm{to}\;\mathrm{face})$$

### 2.12. Physical Properties of Nano-Composite

Physical properties were measured using Equations (5), (7) and (8), respectively. The samples size was 150 mm into 100 mm into 16 mm and a total of three samples of each specimen were tested for each physical property and the average of the four values have been described in the results. The water absorption and thickness swelling tests were performed for 2 h and 24 h.

(5) $$Density=\frac{m}{V}$$

(6) V=Lbt

where, *m* and *V* are mass and volume measured in kg and m^3^ while *b*, *L* and *t* are width, length, and thickness of the specimen, respectively and measured in mm.

(7) $$\mathrm{Water}\;\mathrm{absorption}=\frac{W_{f}-W_{i}}{W_{i}}\times 100$$

where, *W_i_* and *W_f_* are initial and final weights of the specimen, both measured in N, respectively.

(8) $$\mathrm{Thickness}\;\mathrm{swelling}=\frac{T_{f}-T_{i}}{T_{i}}\times 100$$

where, *T_i_* and *T_f_* are initial and final thicknesses of the specimens, respectively and measured in mm.

### 2.13. Statistical Analysis of Nano-Composite

Analysis of variance (ANOVA) with single factor was applied for statistical analysis of results through origin 9, 64-bit software (OriginLab, Northampton, MA, USA).

## 3. Results and Discussion

The results obtained are illustrated in the following paragraphs.

### 3.1. Scanning Electron Microscopy of Cured Urea-Formaldehyde Resin Containing Alumina Nanoparticles

**Figure 4:**
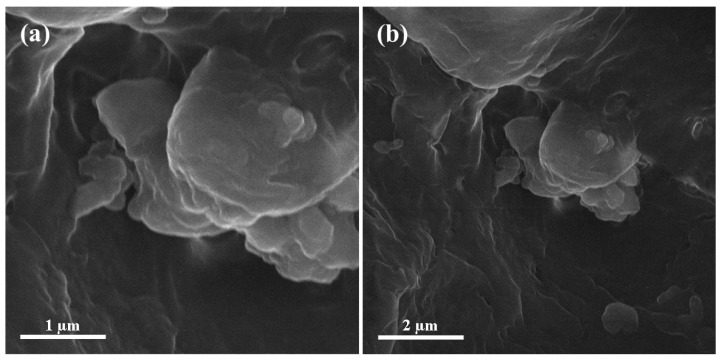
Scanning electron microscopy images of alumina urea-formaldehyde resin (**a**) 50,000× (**b**) 25,000×.

The microstructural analysis and scanning electron microscopy of Al_2_O_3_-UF was conducted for the purpose to examine the role of nanoparticles in the resin. Figure 4 illustrates the scanning electron microscopy images of the urea-formaldehyde resin after curing. An uneven configuration of the resin was found in the cross-linkage. The scanning electron microscopic results clearly identified the partial pits in the urea-formaldehyde resin covered by 3% alumina nanoparticles. Due to robust bonding, minor cracks in the urea-formaldehyde resin were also covered by alumina nanoparticles and hence, an increase in the overall strength of the desired medium density fiberboard has been observed [30]. The bright region in the scanning electron microscopy shows the presence of alumina nanoparticles and the dark region depicts urea-formaldehyde resin. The result was confirmed by Energy Dispersive X-ray Spectroscopy (EDS).

### 3.2. Energy Dispersive X-Ray Spectroscopy (EDS) Analysis

**Figure 5:**
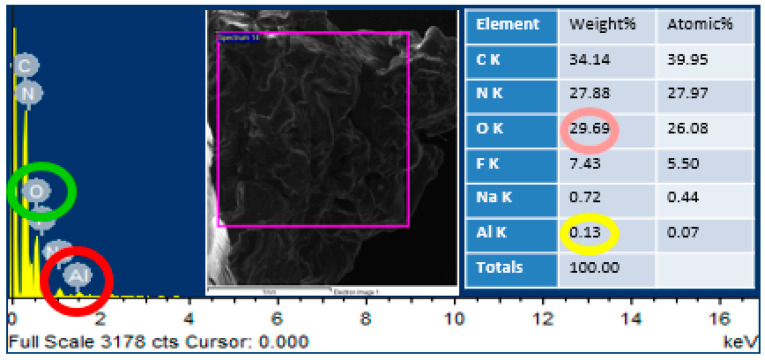
EDS analysis of pure urea-formaldehyde resin.

**Figure 6:**
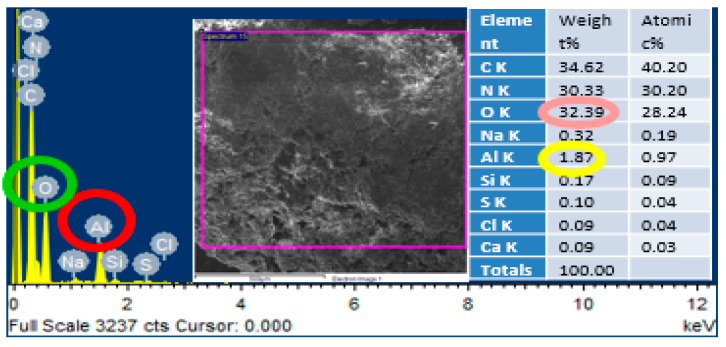
EDS analysis of urea-formaldehyde resin containing 3% alumina nanoparticles.

In order to confirm the presence of alumina nanoparticles in the urea-formaldehyde resin, energy dispersive X-ray spectroscopy was performed on the area mapping of scanning electron microscopic images. One sample with pure urea-formaldehyde resin and another with 3% alumina nanoparticles were selected for energy dispersive x-ray spectroscopy analysis as shown in Figure 5 and Figure 6. In the energy dispersive X-ray spectroscopy analysis, the weight % of oxygen along with potassium (K) and Aluminum (Al) along with potassium (K) was 29.69 and 0.13 for 0% alumina containing UF resin. While these value for 3% alumina containing UF were recorded as 32.39 and 1.87% by weight. Energy peaks correspond to oxygen and aluminum elements in the samples were noted higher than the reference resin.

### 3.3. Fourier Transform Infrared Spectroscopy (FTIR) of Urea-Formaldehyde Resin with and without Alumina Nanoparticles

**Figure 7:**
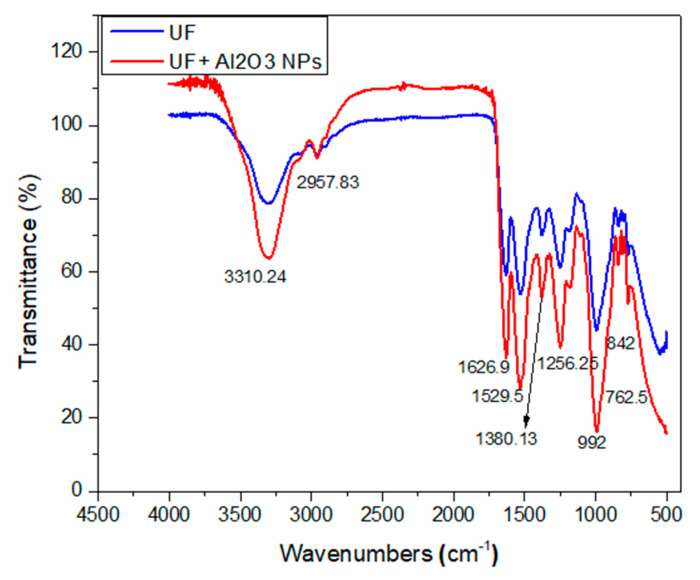
Fourier transform infrared spectroscopy of urea-formaldehyde with or without alumina nanoparticles.

Through this test, a strong adsorption peak at 3310.24 cm^−1^ was noticed in the urea-formaldehyde resin with and without alumina nanoparticles as depicted in Figure 7. With the addition of alumina nanoparticles in the urea-formaldehyde resin, a high peak intensity was observed due the presence of free amino group. The peak values near to 1650 cm^−1^ and 1500 cm^−1^ confirm the presence of C=O, CH_2_OH, amide I and II, and CH_2_OH. The CN group was also observed at peaks 1380.13 cm^−1^ and 1256.25 cm^−1^ [31]. The structural difference was only seen at a peak near to 1000 cm^−1^ which confirms the presence of Al–O bond in 4.5% concentration of the alumina nanoparticles in the urea-formaldehyde resin.

### 3.4. X-Ray Diffraction (XRD) of Cured Urea-Formaldehyde Resin

The X-ray diffraction patterns of urea-formaldehyde resin with 1.5%, 3%, and 4.5% concentration of alumina nanoparticles are shown in Figure 6. The resin was cured at 100 °C before X-ray diffraction. Samples 1, 2, and 3 containing 1.5%, 3%, and 4.5%, Al_2_O_3_ respectively show a peak at 20.6°, 61.25°, and 66.5° with d-spacing of 0.43 nm, 0.151 nm, and 0.141 nm, respectively according to Brag’s Law by using Equation (9).

(9) 2dsinθ=nλ

where, *d* represents the spacing between planes, *θ* shows half of the angle of diffraction, *n* indicates the order of diffraction while *λ* is the wavelength of X-ray. The numerical value of λ is equal to 1.54073 Å.

**Figure 8:**
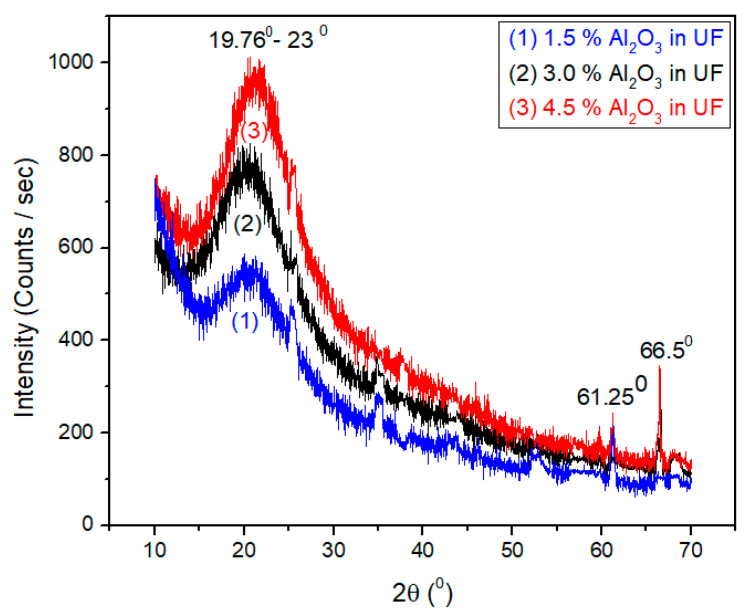
X-ray diffraction of different concentration of alumina nano-particles in urea-formaldehyde resin.

Sample 3 containing 4.5% Al_2_O_3_ shows a peak at 21.25°, 61.25°, and 66.5° with d-spacing of 0.418 nm, 0.151 nm, and 0.141 nm, respectively as can be seen in Figure 8. These results are very close to pure urea-formaldehyde crystal structure as reported by Chen et al. [32]. Hence, we can say that there was no difference among all three samples of cured Al_2_O_3_-UF resin with reference to X-ray diffraction spectrum.

### 3.5. Differential Scan Calorimetry (*DSC*) of Urea-Formaldehyde with and without Alumina Nanoparticles

**Figure 9:**
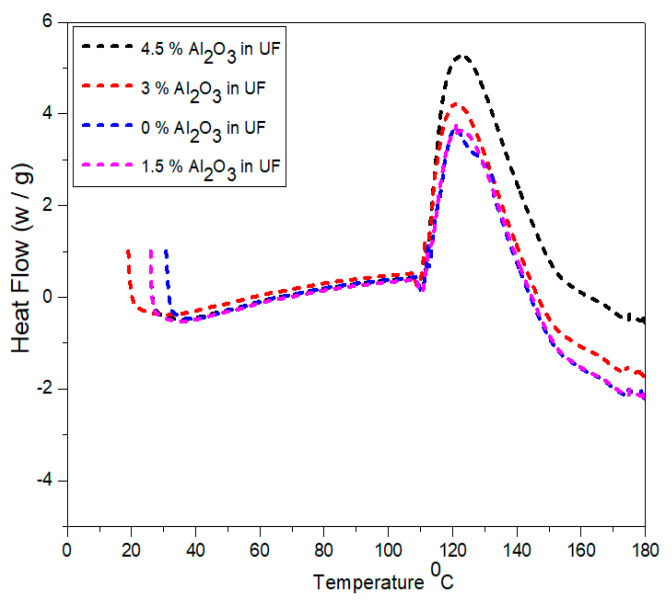
Differential scan calorimetry of urea-formaldehyde resin with different concentrations of alumina nanoparticles.

Figure 9 represents the temperature ranges versus heat flow curves for the selected four samples containing urea-formaldehyde and alumina urea-formaldehyde resin. The curves indicate that with the addition of different concentrations of alumina nanoparticles, the curing temperature decreases. The total heat content increases with the concentartion of alumina nanoparticles. The peak at 120 °C in 1.5% alumina is formed due to formation of bonding in urea-formaldehyde at the interface. The alumina nanoparticles has adopted an effect analogous to former thermosetting resin. As studied in literature, alumina nanoparticles delivered Lewis acidity. Because of the presence of hydoxyl groups in alumina nanoparticles, the superficial behaved as a catalytic agent and polymerized the urea-formadehyde resin as reported by Kumar et al. [33].

### 3.6. Thermo-Gravimetric Analysis (*TGA*) of Urea-Formaldehyde with and without Alumina Nanoparticles

**Figure 10:**
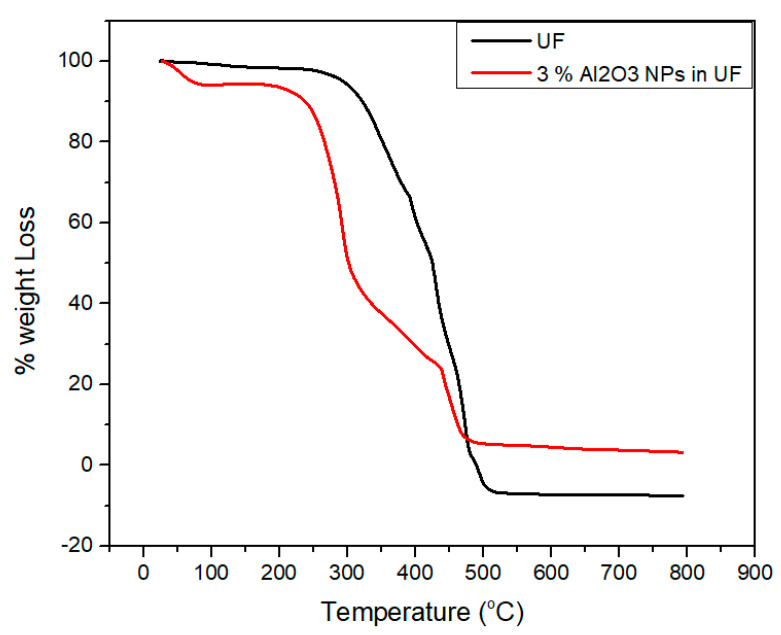
Thermo-gravimetric analysis of urea-formaldehyde resin with and without alumina nanoparticles.

The curves of the weight losses versus temperature are shown in Figure 10. As the temperature rises from 20 °C and reaches to 140 °C, minor changes in weight loss has been observed due to the moisture absorption and dehydration as investigated by Franceschi et al. [34].

Due to the degradation of urea-formaldehyde resin, considerable weight loss has been observed because of the presence of intra and intermolecular carbon and hydrogen bonds. This phenomenon might also be happen due to the random cleavage of nitrogen bonds in the urea-formaldehyde resin.

After the alkaline hydrolysis, the functional groups become active which result in more moisture content in the urea-formaldehyde resin as compared to concentrated resin. By the addition of alumina nanoparticles, a strong bonding and van der Waals forces are formed among the functional groups of concentrated resin leading to high thermal stability. The degradation happens within a temperature ranging from 230 °C to 500 °C. The concentrated resin is hydrolyzed and its intermolecular interface converts tough which ultimately increase the peak zone.

### 3.7. Scanning Electron Microscopy (SEM) of Final Medium Density Fiberboard

**Figure 11:**
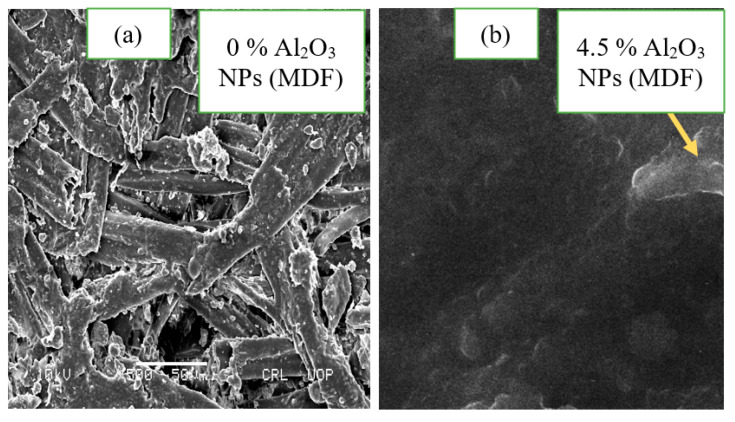
Scanning electron microscopic images of (**a**) medium density fiberboard without urea-formaldehyde and (**b**) with urea-formaldehyde.

The final medium density fiberboard with and without alumina nanoparticles is shown in Figure 11. Scanning electron microscopy reveals the cluster structure due to alumina nanoparticles using sodium montmorillonite nanoclay [35]. Visible voids were observed in the final medium density fiberboard made of pure urea-formaldehyde resin. These voids were covered by alumina nanoparticles because of the discrete and head-to-head regions.

### 3.8. Statistical Analysis of the Mechanical and Physical Properties of MDF

**Figure 12:**
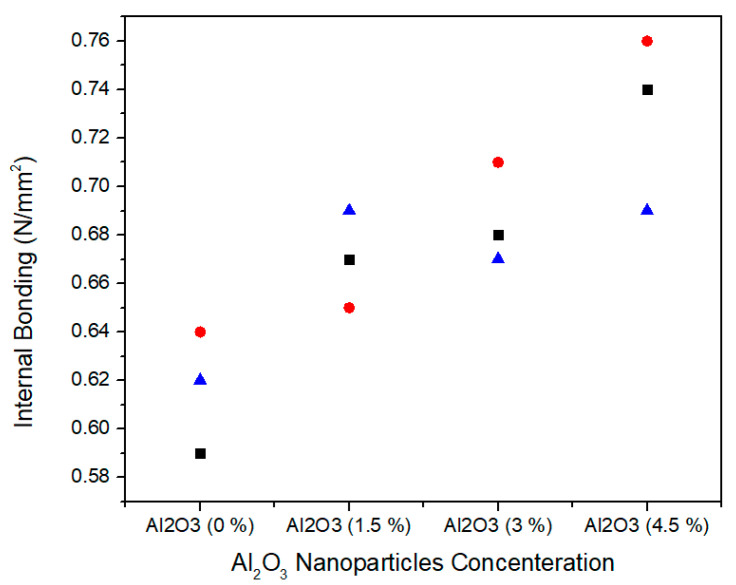
Numerical values of internal bonding of various treatments of alumina nanoparticles.

The internal bonding is determined by tensile strength of the MDF. Figure 12 shows the single factor ANOVA results of three treatments comparison of internal bonding values for 0.0%, 1.5%, 3.0%, and 4.5% concentration levels of Al_2_O_3_ nanoparticles. For 0.0% alumina, the three treatments values of internal bonding are 0.59, 0.64, and 0.64 N/mm^2^. For 1.5% Alumina the three treatments values are 0.67, 0.65, and 0.69 N/mm^2^. Similarly, for 3.0% alumina nanoparticles, all the three counts have the values of 0.68, 0.71, and 0.67 N/mm^2^ internal bonding values. As the concentration level increase from 3.0% to 4.5%, the internal bonding values 0.74, 0.76, and 0.69 N/mm^2^ increase for all treatments. Red circle, blue triangle, and black square are iterations for each concentration of alumina nanoparticles.

Table 3 summarizes the ANOVA statistical approach for three treatments of 0.0%, 1.5%, 3.0%, and 4.5% alumina nanoparticles. 0.0% alumina containing medium density fiberboard has a mean value of 0.61 N/mm^2^ and variance 0.000633. While 1.5%, 3.0%, and 4.5% alumina containing medium density fiberboard have 0.67, 0.68 and 0.73 mean values with variances 0.0004, 0.00043, and 0.0013, respectively. These internal bonding values are statistically altered from each other and the single factor ANOVA consequences confirm that the probability (*p*-value) is 0.005119869.

**Figure 13:**
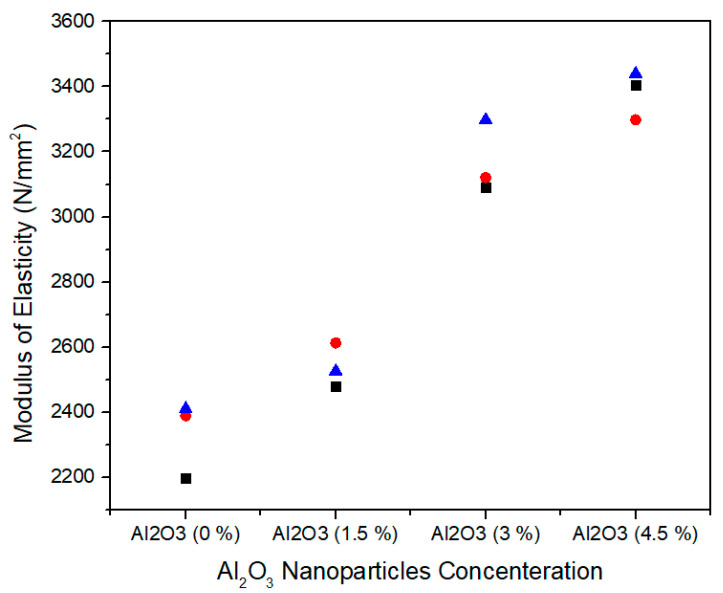
Numerical values of modulus of elasticity (MOE) of various treatments of alumina nanoparticles.

The modulus of elasticity is normally measured the medium density fiberboard resistance to being deformed elastically when a stress is applied on it. Figure 13 shows the single factor ANOVA results of three treatments comparison for 0.0%, 1.5%, 3.0%, and 4.5% concentration levels of Al_2_O_3_ nanoparticles. For 0.0% alumina, the three treatments values of modulus of elasticity are 2197.29, 2388.51, and 2410.46 N/mm^2^. For 1.5% alumina the three treatments values are 2480.16, 2612.78, and 2526.10 N/mm^2^. In the same way, for 3.0% alumina nanoparticles, all the three counts have 3091.50, 3120.32, and 3205.45 N/mm^2^ modulus of elasticity values. As the concentration level increases from 3.0% to 4.5% the modulus of elasticity values 3404.53, 3298.21, and 3440.33 N/mm^2^ show an increase for all treatments.

Table 4 summarizes the ANOVA statistical approach of modulus of elasticity values for three the treatments of 0.0%, 1.5%, 3.0%, and 4.5% alumina nanoparticles. A 0.0% alumina containing medium density fiberboard has a mean value of 2332.087 N/mm^2^ and a variance of 13,748.06. While 1.5%, 3.0%, and 4.5% alumina containing medium density fiberboard have 2539.68, 3139.09, and 3381.023 modulus of elasticity mean values with variance 0.4535.328, 3510.385, and 5463.946, respectively. These modulus of elasticity values are statistically altered from each other and the single factor ANOVA consequences confirm that the probability (*p*-value) is 8.4772 × 10^−7^.

**Figure 14:**
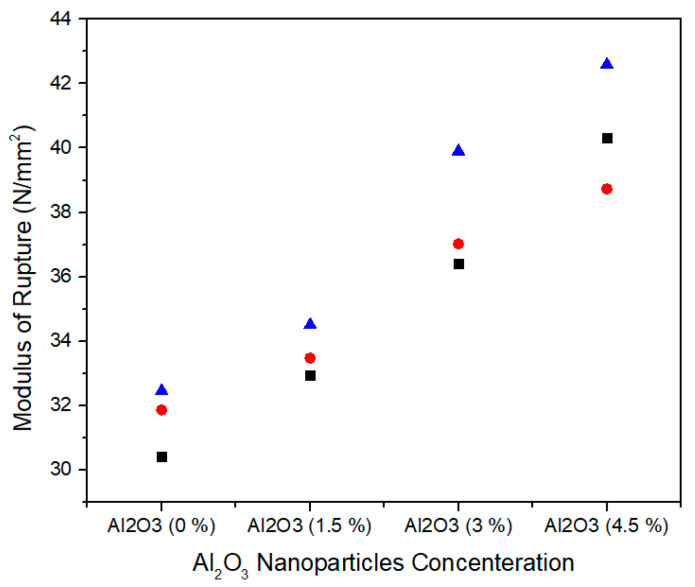
Numerical values of modulus of rupture (MOR) of various treatments of alumina nanoparticles.

The modulus of rupture represents the flexural strength of medium density fiberboard and is defined as the stress just before yield. Figure 14 shows the single factor ANOVA results of three treatments comparison for 0.0%, 1.5%, 3.0%, and 4.5% concentration levels of Al_2_O_3_ nanoparticles. For 0.0% alumina, the three treatments values of modulus of rupture are 30.42, 31.86, and 32.45 N/mm^2^ while for 1.5% alumina the three treatments values of modulus of rupture are 32.94, 33.47 and 34.50 N/mm^2^. Similarly, for 3.0% alumina nanoparticles, all the three counts have 36.41, 37.02, and 39.90 N/mm^2^ modulus of rupture values. As the concentration level increase from 3.0% to 4.5%, the modulus of rupture values 40.32, 38.73, and 42.59 N/mm^2^ show as significant increase for all treatments.

Table 5 summarizes the ANOVA statistical approach of modulus of rupture values for the three treatments of 0.0%, 1.5%, 3.0%, and 4.5% alumina nanoparticles. 0.0% alumina containing medium density fiberboard has modulus of rupture mean value of 31.57 N/mm^2^ and variance of 1.09 while 1.5%, 3.0%, and 4.5% alumina containing medium density fiberboard have 33.63, 37.77, and 40.54 modulus of rupture mean values with variance 0.629, 3.474, and 3.76, respectively. These modulus of rupture values are statistically altered from each other and the single factor ANOVA consequences confirm that the probability (*p*-value) is 0.000329.

**Figure 15:**
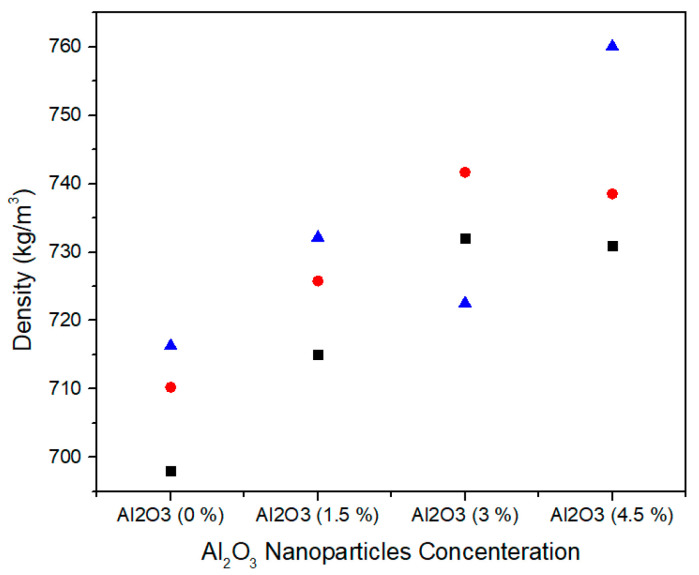
Numerical values of density of various treatments of alumina nanoparticles.

Density represents the mass per unit volume of medium density fiberboard. Figure 15 shows the single factor ANOVA results of three treatments comparison of density for 0.0%, 1.5%, 3.0%, and 4.5% concentration levels of alumina nanoparticles. For 0.0% alumina, the three treatments values of density are 698.05, 710.24, and 716.29 kg/m^3^. For 1.5% alumina the three treatments values of density are 715, 725.8, and 732.15 kg/m^3^. Likewise, for 3.0% alumina nanoparticles, all the three counts have 732, 741.68, and 722.50 N/mm^2^ density values. As the concentration level increase from 3.0% to 4.5%, the density values 730.91, 738.54, and 760.06 kg/m^3^ show significant increase for all treatments.

Table 6 summarizes the ANOVA statistical approach of density values for three treatments of 0.0%, 1.5%, 3.0%, and 4.5% alumina nanoparticles. The 0.0% alumina containing medium density fiberboard has density mean value of 708.19 kg/m^3^ and variance of 86.31. While 1.5%, 3.0%, and 4.5% alumina containing medium density fiberboard have 724.3, 732.06, and 743.17 density mean values with variance 75.18, 91.97, and 228.5, respectively. These density values are statistically altered from each other and the single factor ANOVA consequences confirm that the probability (*p*-value) is 0.025461.

**Figure 16:**
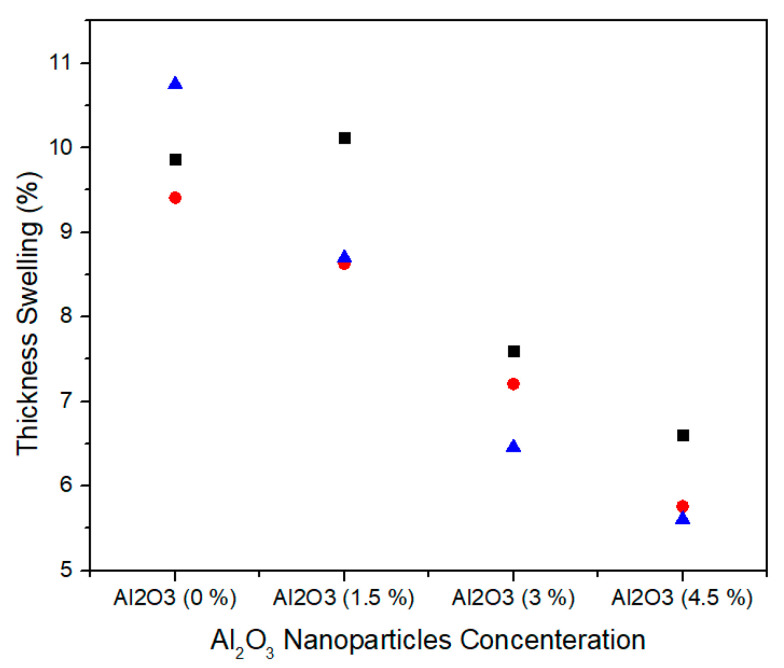
Numerical values of Thickness Swelling of various treatments of Alumina nanoparticles.

Thickness swelling represents the stability performance of medium density fiberboard. Figure 16 shows the single factor ANOVA results of three treatments comparison of thickness swelling for 0.0%, 1.5%, 3.0%, and 4.5% concentration levels of alumina nanoparticles. For 0.0% alumina, the three treatments values of thickness swelling are 9.87, 9.41, and 10.75% and for 1.5% alumina the three treatments values of thickness swelling are 10.12, 8.63 and 8.70%. Similarly, for 3.0% alumina nanoparticles, all the three counts have 7.60, 7.21, and 6.46% thickness swelling values. It can be noted that as the concentration level increases from 3.0% to 4.5%, the thickness swelling values 6.60, 5.76, and 5.61% show significant decrease for all treatments.

Table 7 summarizes the ANOVA statistical approach of thickness swelling values for three treatments of 0.0%, 1.5%, 3.0% and 4.5% alumina nanoparticles. 0.0% alumina containing medium density fiberboard has thickness swelling mean value of 10.01% and variance of 0.46. While 1.5%, 3.0%, and 4.5% alumina containing medium density fiberboard have 9.15, 7.09, and 5.99 thickness swelling mean values with variances 0.70, 0.33, and 0.28, respectively. These thickness swelling values are statistically altered from each other and the single factor ANOVA consequences confirm that the probability (*p*-value) is 0.000282.

**Figure 17:**
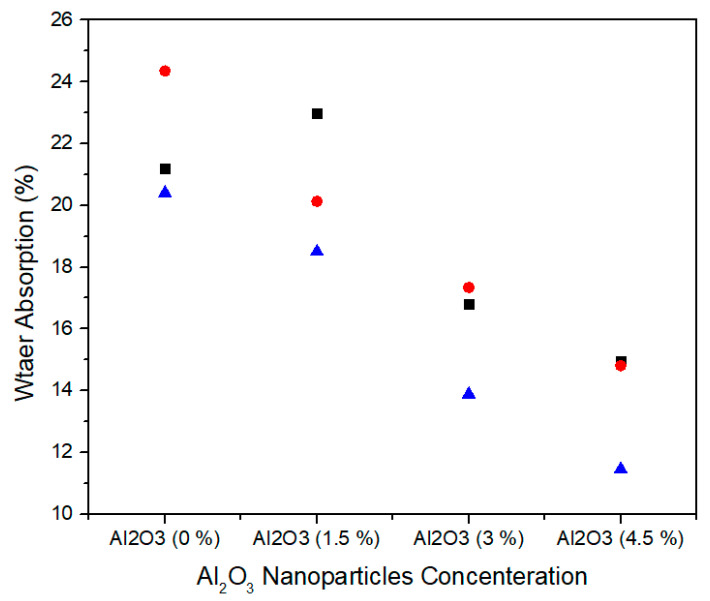
Numerical values of water absorption of various treatments of alumina nanoparticles.

The water absorption is the ability of medium density fiberboard to absorb water when immersed in it. Figure 17 shows the single factor ANOVA results of three treatments comparison of water absorption for 0.0%, 1.5%, 3.0%, and 4.5% concentration levels of alumina nanoparticles. For 0.0% alumina, the three treatments values of water absorption are 21.20, 24.35, and 20.40% and for 1.5% alumina, the three treatments values of water absorption are 22.97, 20.13, and 18.50%. Similarly, for 3.0% alumina nanoparticles, all the three counts have 16.80, 17.34, and 13.88% water absorption values. As the concentration level increase from 3.0% to 4.5% the water absorption values 14.95, 14.81, and 11.45% show significant decrease for all treatments.

Table 8 summarizes the ANOVA statistical approach of water absorption values for the three treatments of 0.0%, 1.5%, 3.0%, and 4.5% alumina nanoparticles. The 0.0% alumina containing medium density fiberboard has water absorption mean value of 21.98% and variance of 4.36. While 1.5%, 3.0%, and 4.5% alumina containing medium density fiber board have 20.53, 16.00, and 13.73 water absorption mean values with variance 5.11, 3.46, and 3.92, respectively. These thickness swelling values are statistically altered from each other and the single factor ANOVA consequences confirm that the probability (*p*-value) is 0.003754.

### 3.9. Final Properties of Medium Density Fiberboard

The physical and mechanical properties of medium density fiberboard samples have been investigated using 0%, 1.5%, 3%, and 4.5% of alumina nanoparticles and urea-formaldehyde resin. Each sample was tested for three iterations and the average value of each property under specific configuration was determined. The mechanical properties such as internal bonding, modulus of rupture and modulus of elasticity were tested using WDW-30 (Electromechanical Universal Testing Machine of JINAN Precision Testing Equipment Company Limited, Jinan, China). Each sample was tested for mechanical properties under the specific configuration of alumina nanoparticles as summarized in Table 9.

During the hot pressing, the resin is bonded with wood fibers in cross-link pattern which refers to internal bonding. It is clear that at zero concentration level of alumina nanoparticle, the value of internal bonding resulted in 0.61 N/mm^2^, which indicates the reference for all other configurations of alumina nanoparticles in urea-formaldehyde resin. With the addition of 1.5%, 3.0%, and 4.5% of alumina nanoparticles in the urea-formaldehyde resin increased the internal bonding by 5.97%, 10.29%, and 16.4%, respectively and is also meet with EN-319 [36] standards. Hence, the internal bonding values increased linearly with the increase in the concentration of alumina nanofillers.

The reason of linear relationship between internal bonding and alumina nanofillers is due to increase in the cross-link density of urea-formaldehyde resin. Further, alumina nanoparticles also have the capability of high heat transfer which leads to fast curing of urea-formaldehyde resin, reduced press time and increased production.

It can be noted that the modulus of elasticity value at 0% alumina nanoparticle in urea-formaldehyde resin in Table 4 is 2332.08 N/mm^2^. When it is compared with 1.5%, 3%, and 4.5% concentrations, the corresponding modulus of elasticity values increased by 8.17%, 25.70%, and 31% respectively and is also meet with EN-310 [37] standard values.

The modulus of rupture values increases with the increase in concentration of alumina nanoparticles. The increase in nanofillers concentration into 1.5%, 3%, and 4.5% leaded to increased modulus of rupture values by 6.52%, 16.4%, and 22.12%, respectively when compared to medium density fiberboard containing 0% nanofillers and also meet with EN-310 [37] standard values. The reason of significant increase in modulus of rupture values is due to fast curing of the nanofillers.

The physical properties such as density, thickness swelling and water absorption are summarized in Table 10. The samples were tested for 0%, 1.5%, 3%, and 4.5% concentration levels of alumina nanoparticles with three iterations of each sample and the average values were taken into consideration. Both thickness swelling and water absorption tests were performed for 24 h according to British Standard EN-317 1993 [38] and ASTM D570 [39] respectively.

The density increases with the increase in the concentration of nanofillers due to increase in the mass of the nanofillers. A gradual reduction in the thickness swelling values of the samples for 24 h was observed which is due to reduction of pores in the MDF panels. Similarly, the water absorption values also decrease with the increase in concentration of nanofillers which is due to better curing of the panels during hot pressing.

## 4. Conclusions

In this research, the impact of alumina nanoparticles on the properties of medium density fiberboard under fixed process conditions has been investigated. It has been observed that the physical and mechanical properties of the medium density fiberboard composites have been improved significantly the nonlinear relation in case of modulus of elasticity. For 4.5% concentration level of alumina, the internal bond, modulus of elasticity and modulus of rupture values increased to 16.4%, 31%, and 22.12% respectively. The thickness swelling and water absorption for 24 h increased up to 40.15% and 37.53%, respectively when compared to normal medium density fiberboard. Hence, with the increase in concentration of nanoparticles, the physical and mechanical properties improve considerably. The results of the structure morphology showed that alumina nanoparticles can fill the partial pit created in fiber matrix. Therefore, to increase the strength of medium density fiberboard, it is strongly recommended to use alumina nanoparticles in urea-formaldehyde resin.

A hypothesis can be made for hybrid approach to get better results in which grapheme, carbon nanotubes and activated charcoal can be mixed with alumina nanoparticles.

## Figures and Tables

**Table 1:** Specifications of urea-formaldehyde.

Viscosity	Density	pH	Free Formaldehyde	Gel Time	Solid Content
230–260	1.22	9	0.75	53	50

**Table 2:** Composition of Al_2_O_3_-UF nanofillers.

Materials	Composition			
	Al_1_	Al_2_	Al_3_	Al_4_
UF	195	195	195	195
Al_2_O_3_	0	3	6	9

**Table 3:** Internal Bonding values of Al_2_O_3_-UF MDF for various treatments.

	**Groups**	**Count**	**Sum**	**Average (IB)**	**Variance**	
	Al_2_O_3_ (0.0%)	3	1.85	0.616666667	0.000633333	
	Al_2_O_3_ (1.5%)	3	2.01	0.670000000	0.000400000	
	Al_2_O_3_ (3.0%)	3	2.06	0.686666667	0.000433333	
	Al_2_O_3_ (4.5%)	3	2.19	0.730000000	0.001300000	
**ANOVA**						
**Source of Variation**	**SS**	**df**	**MS**	**F**	***p*-value**	**F crit**
Between Groups	0.019758	3	0.006586111	9.522088353	0.005119869	4.066180551
Within Groups	0.005533	8	0.000691667			
Total	0.025292	11				

**Table 4:** MOE values of Al_2_O_3_-UF MDF for various treatments.

	**Groups**	**Count**	**Sum**	**Average**	**Variance**	
	Al_2_O_3_ (0.0%)	3	6996.260	2332.087	13,748.06	
	Al_2_O_3_ (1.5%)	3	7619.040	2539.680	4535.328	
	Al_2_O_3_ (3.0%)	3	9417.270	3139.090	3510.385	
	Al_2_O_3_ (4.5%)	3	10,143.07	3381.023	5463.946	
**ANOVA**						
**Source of Variation**	**SS**	**df**	**MS**	**F**	***p*-value**	**F crit**
Between Groups	2,190,225	3	730,075	107.1366	8.4772 × 10^−7^	4.066181
Within Groups	54,515.43	8	6814.429			
Total	2,244,741	11				

**Table 5:** MOR values of Al_2_O_3_-UF MDF for various treatments.

	**Groups**	**Count**	**Sum**	**Average**	**Variance**	
	Al_2_O_3_ (0.0%)	3	94.730	31.57667	1.090433	
	Al_2_O_3_ (1.5%)	3	100.91	33.63667	0.629233	
	Al_2_O_3_ (3.0%)	3	113.33	37.77667	3.474433	
	Al_2_O_3_ (4.5%)	3	121.64	40.54667	3.763433	
**ANOVA**						
**Source of Variation**	**SS**	**df**	**MS**	**F**	***p*-value**	**F crit**
Between Groups	146.7788	3	48.92628	21.8481	0.000329	4.066181
Within Groups	17.91507	8	2.239383			
Total	164.6939	11				

**Table 6:** Density values of Al_2_O_3_-UF MDF for various treatments.

	**Groups**	**Count**	**Sum**	**Average**	**Variance**	
	Al_2_O_3_ (0.0%)	3	2124.58	708.1933	86.31603	
	Al_2_O_3_ (1.5%)	3	2172.95	724.3167	75.18083	
	Al_2_O_3_ (3.0%)	3	2196.18	732.0600	91.97080	
	Al_2_O_3_ (4.5%)	3	2229.51	743.1700	228.5083	
**ANOVA**						
**Source of Variation**	**SS**	**df**	**MS**	**F**	***p*-value**	**F crit**
Between Groups	1943.840	3	647.9466	5.377418	0.025461	4.066181
Within Groups	963.9519	8	120.4940			
Total	2907.792	11				

**Table 7:** Thickness swelling values of Al_2_O_3_-UF MDF for various treatments.

	**Groups**	**Count**	**Sum**	**Average**	**Variance**	
	Al_2_O_3_ (0.0%)	3	30.03	10.01	0.4636	
	Al_2_O_3_ (1.5%)	3	27.45	09.15	0.7069	
	Al_2_O_3_ (3.0%)	3	21.27	07.09	0.3357	
	Al_2_O_3_ (4.5%)	3	17.97	05.99	0.2847	
**ANOVA**						
**Source of Variation**	**SS**	**df**	**MS**	**F**	***p*-value**	**F crit**
Between Groups	30.6492	03	10.21640	22.81847	0.000282	4.066181
Within Groups	3.58180	08	0.447725			
Total	34.2310	11				

**Table 8:** Water Absorption values of Al_2_O_3_-UF MDF for various treatments.

	**Groups**	**Count**	**Sum**	**Average**	**Variance**	
	Al_2_O_3_ (0.0%)	3	65.95	21.98333	4.360833	
	Al_2_O_3_ (1.5%)	3	61.60	20.53333	5.117233	
	Al_2_O_3_ (3.0%)	3	48.02	16.00667	3.464933	
	Al_2_O_3_ (4.5%)	3	41.21	13.73667	3.926533	
**ANOVA**						
**Source of Variation**	**SS**	**df**	**MS**	**F**	***p*-value**	**F crit**
Between Groups	133.2516	3	44.41721	10.53194	0.003754	4.066181
Within Groups	33.73907	8	4.217383			
Total	166.9907	11				

**Table 9:** The Final mechanical properties of 16 mm MDF samples for different concentrations of alumina nanoparticles.

MDF Specimen	Internal Bonding	Modulus of Elasticity	Modulus of Rupture
S_0.0_Al_0.0_	0.61	2332.08	31.57
S_1.5_Al_1.5_	0.67	2539.68	33.63
S_3.0_Al_3.0_	0.68	3139.09	37.77
S_4.5_Al_4.5_	0.73	3381.02	40.54
EN Standards	0.7 ± 0.03	≥2800	≥25

Internal Bonding EN-319 [36], MoE and MoR EN-310 [37].

**Table 10:** The final physical properties of 16 mm MDF samples for different concentrations of alumina nanoparticles.

MDF Specimen	Density (kg/m^3^)	TS *	WA *
S_0.0_Al_0.0_	708	10.01	21.98
S_1.5_Al_1.5_	724	9.15	20.53
S_3.0_Al_3.0_	732	7.09	16.00
S_4.5_Al_4.5_	743	5.99	13.73
Standard	720 ± 20	≤12	<45

* 24 h TS (EN-317 standard) [38], WA (ASTM D570 standard) [39], Density (EN-323 standard) [40].
